# Prediction of outpatients with conjunctivitis in Xinjiang based on LSTM and GRU models

**DOI:** 10.1371/journal.pone.0290541

**Published:** 2023-09-21

**Authors:** Yijia Wang, Xianglong Yi, Mei Luo, Zhe Wang, Long Qin, Xijian Hu, Kai Wang

**Affiliations:** 1 College of Mathematics and System Science, Xinjiang University, Urumqi Xinjiang, China; 2 Department of Ophthalmology, The First Affiliated Hospital of Xinjiang Medical University, Urumqi, Xinjiang, China; 3 Department of Medical Information, Zhongshan School of Medicine, Sun Yat-sen University, Guangzhou, China; 4 EClinCloud (Shenzhen) Technology Co., Ltd, Shenzhen Bay Science and Technology Ecological Park, Nanshan District, Shenzhen, Guangdong, China; 5 Department of Medical Engineering and Technology, Xinjiang Medical University, Urumqi Xinjiang, China; UCSI University Kuala Lumpur Campus: UCSI University, MALAYSIA

## Abstract

**Background:**

Reasonable and accurate forecasting of outpatient visits helps hospital managers optimize the allocation of medical resources, facilitates fine hospital management, and is of great significance in improving hospital efficiency and treatment capacity.

**Methods:**

Based on conjunctivitis outpatient data from the First Affiliated Hospital of Xinjiang Medical University Ophthalmology from 2017/1/1 to 2019/12/31, this paper built and evaluated Long Short-Term Memory (LSTM) and Gated Recurrent Unit (GRU) models for outpatient visits prediction.

**Results:**

In predicting the number of conjunctivitis visits over the next 31 days, the LSTM model had a root mean square error (RMSE) of 2.86 and a mean absolute error (MAE) of 2.39, the GRU model has an RMSE of 2.60 and an MAE of 1.99.

**Conclusions:**

The GRU method can better predict trends in hospital outpatient flow over time, thus providing decision support for medical staff and outpatient management.

## 1. Introduction

Conjunctivitis is a general term for an acute or chronic inflammatory reaction in the conjunctival tissue caused by various causes. Conjunctivitis is the most common eye disease, as well as one of the causes of serious health and economic burdens worldwide. It is caused mainly by various viral, bacterial, or allergic substances as well as by self-inflammatory allergies [1]. Its sufferers are usually contagious, and outbreaks of infectious diseases can lead to serious morbidity. Due to the increase in the number of patients and the growing complexity of their health conditions, the pressure on the outpatient department, which is the hospital’s external service window, increases every year [2]. The hospital outpatient department is an important part of the hospital organization and has the function of diagnosing, treating, and protecting the health of patients. Thus, forecasting outpatient visits is a necessity for hospital management. Accurate forecasting of outpatient visits can improve outpatient services, make more efficient use of assets, plan operations more effectively, reduce costs and increase revenue [3].

A number of studies have proposed different prediction methods for conjunctivitis. Seo et al. [4] developed a multi-level prediction model to predict conjunctivitis outpatient rates in Korea. Youn et al. [5] used a general regression model to predict the incidence of Dry Eye Syndrome (DES) in South Korea. Qiu et al. [6] used the exponential smoothing model and the seasonal autoregressive integrated moving average (SARIMA) model to analyze and predict acute hemorrhagic conjunctivitis(AHC) incidence in Chongqing. Liu et al. [7] constructed SARIMA and exponential smoothing (ETS) models to predict the trend in incidence in mainland China and provided evidence for the government to formulate policies regarding acute hemorrhagic conjunctivitis (AHC) prevention. With the development of artificial intelligence (AI), machine learning algorithms have shown their advantages in predictions and recognitions [8–10]. Chen et al. [11] compared the ability of seven machine learning methods to predict the number of patients due to conjunctivitis (Lasso penalized linear model, Decision tree, Boosting regression, Bagging regression, Random forest, Support vector, and Neural network). These models cannot describe the stochastic and non-linear nature of outpatient volumes, and they do not better simulate and capture the non-linear characteristics of the data when dealing with non-linear data, resulting in less stable prediction results [12,13].

To overcome the limitations mentioned above, a number of methods related to deep learning have been used by many researchers to build predictive models. Many studies have compared deep learning methods with traditional methods and they have found that deep learning methods are more accurate than traditional methods [14–16]. In deep learning, Recurrent Neural Networks (RNN) have been introduced [17]. However, a long-term dependency caused by the vanishing or explosive gradient problem cannot be handled by RNN [18]. Therefore, the Long Short-Term Memory (LSTM) method is proposed. This method adds gating structures and memory units to the RNN, allowing the network to decide the information to forget and the information to propagate backward. It has the ability to resolve gradient explosion and gradient disappearance [19]. In recent years, LSTM has been quietly applied in many fields, such as stock prices [20], speech recognition [21], and disease prediction (e.g. hand, foot and mouth disease [12,15], COVID-19 [16], and HIV [22]).

GRU networks are proposed on the basis of LSTM networks, which also take into account long-term dependencies. Compared to the LSTM, the GRU has one less gate function and requires fewer parameters, therefore a shorter training time is required [23]. There are similarities and differences between LSTM and GRU methods, but it is impossible to judge theoretically which method is better. Bahdanau et al [24] showed that based on their preliminary experiments on machine translation, the two methods performed comparably. However, it is not known whether this task applies to other areas, so many scholars have made empirical comparisons, such as stock prediction [25], traffic flow prediction [26], short-term runoff prediction [27], and temperature forecast [28].

To date, there is literature on the use of LSTM for disease prediction, but there has not been a comparison of LSTM and GRU models to study the number of conjunctivitis patients. This study intends to identify the best model for predicting the number of patients due to conjunctivitis in Xinjiang by comparing the LSTM and GRU models, based on the daily incidence of conjunctivitis from 2017 to 2019 in Xinjiang.

The paper is structured as follows. Section 2 includes the theoretical background, which describes the methods involved in this paper. Then, the results and discussion are shown in Sections 3 and 4, respectively.

## 2. Materials & methods

### 2.1 Data collection

**Fig 1:**
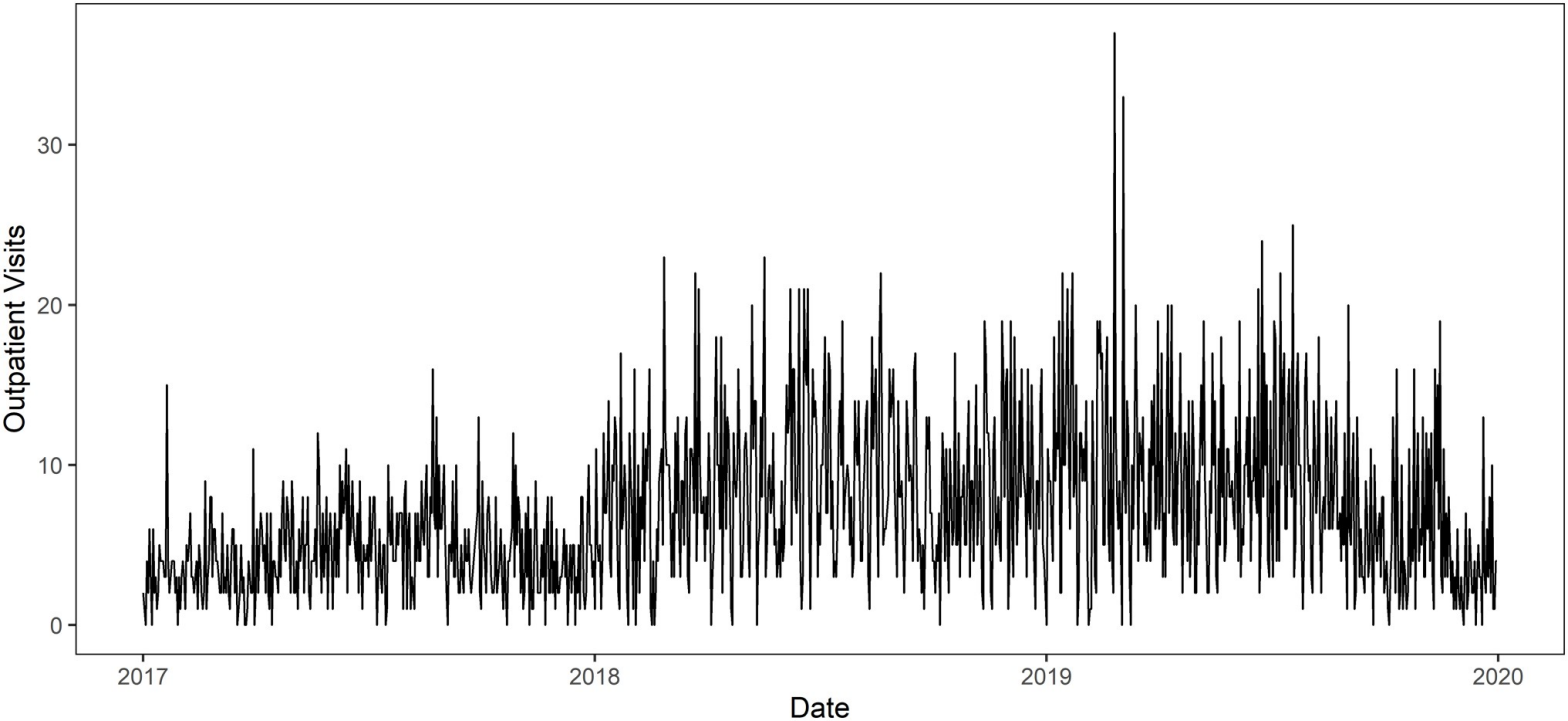
Volume of each day’s conjunctivitis clinic from January 1, 2017 to December 31, 2019.

Data on outpatient visits for conjunctivitis from January 1, 2017, to December 31, 2019, were collected from the Department of Ophthalmology of The First Affiliated Hospital of Xinjiang Medical University, which is one of the largest ophthalmology clinics in Xinjiang Uyghur Autonomous Region. Information on patients with conjunctivitis (non-specific) was collected daily, in addition to visits to the emergency department, and there are no missing data (Fig 1). The data are true and reliable.

This study was approved by the Ethics Committee of the First Affiliated Hospital of Xinjiang Medical University (K202205-08) and followed the tenets of the Declaration of Helsinki.

### 2.2 model and method

#### 2.2.1 LSTM model

**Fig 2:**
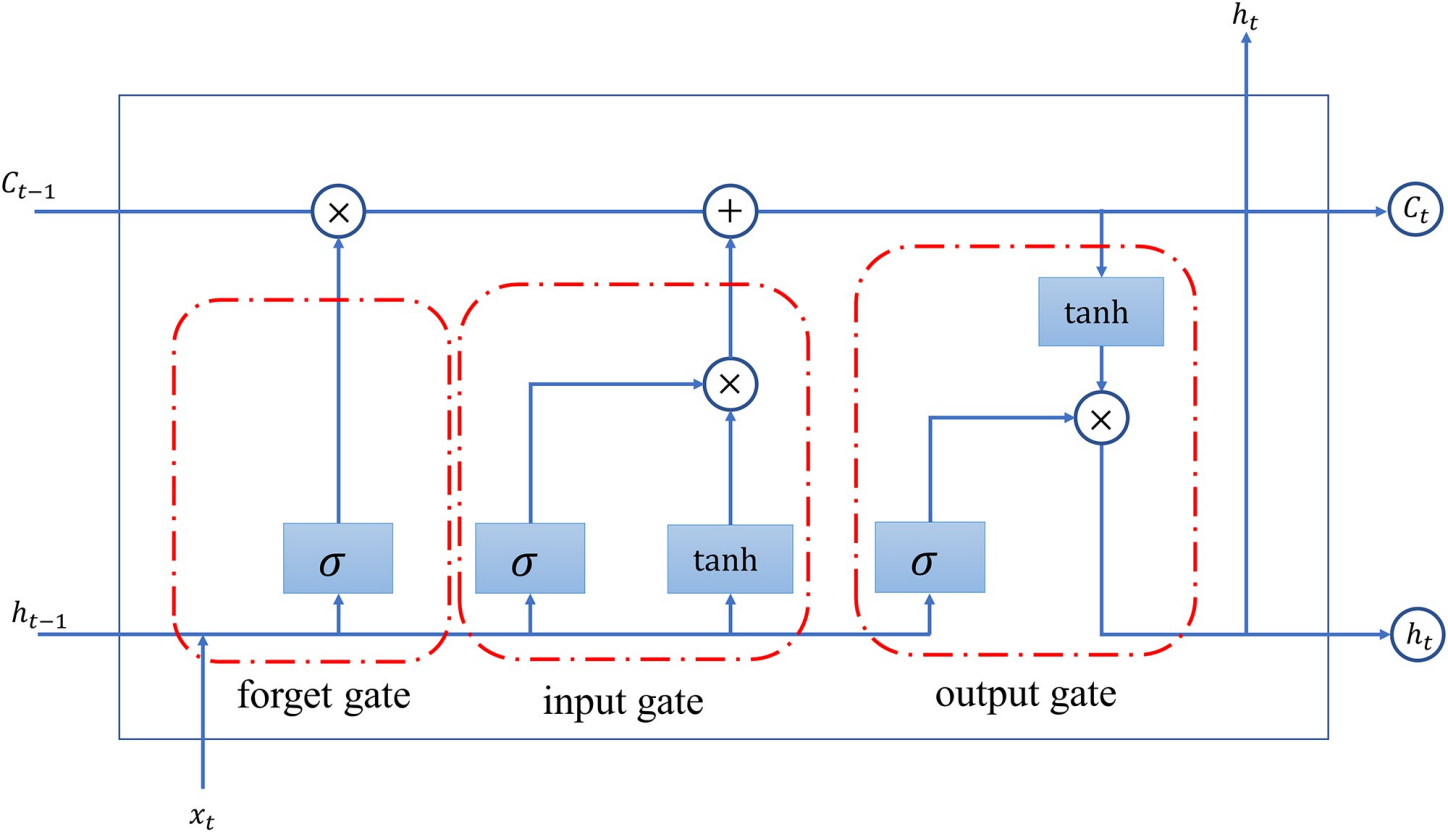
The structure of LSTM cell.

The LSTM model takes into account the continuity of the time series and can effectively solve the problem of gradient disappearance during the gradient descent of the neural network. It uses forgetting gates, input and output gates to filter and control incoming information, and introduces "memory cell states" to store information for long periods of time. Its structure is shown in Fig 2. More detailed introduction of LSTM algorithm can be found in Hochreiter et al [29].

The training and prediction of the LSTM model can be divided into three steps. First, the data is normalized. Next, a two-layer stacked LSTM structure was built. The neuron options for the hidden layer are 8/16/32/64/72/128. Adaptive Moment Estimation (Adam), Stochastic Gradient Descent (SGD), and Root Mean Square Prop (RMSprop) are optional optimization functions. The epochs are set at 50,100,150,200 or 250 during the learning process. The best model was selected based on the minimum RMSE of the test set.

#### 2.2.2 GRU model

**Fig 3:**
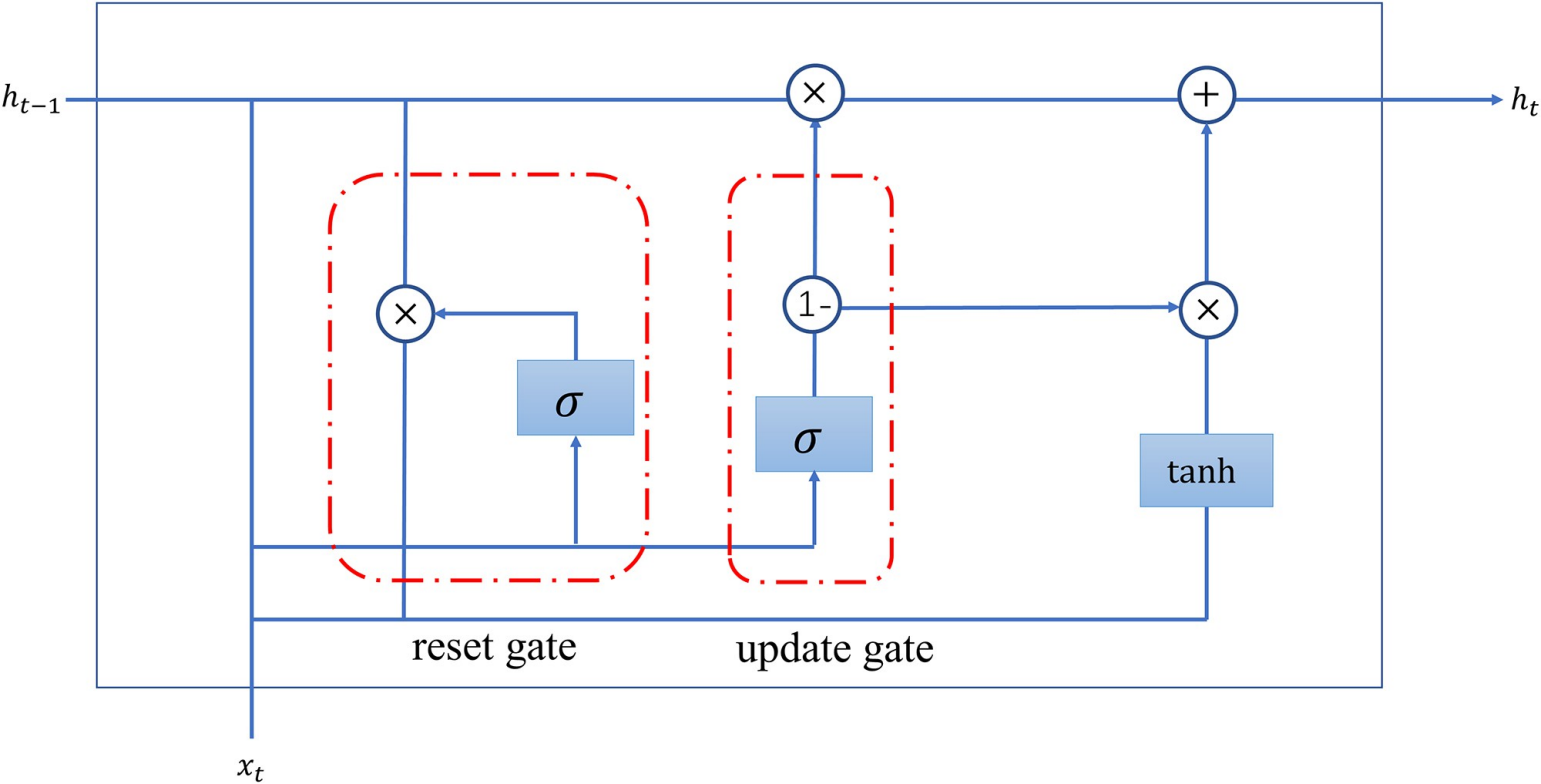
The structure of GRU cell.

The GRU neural network and the LSTM neural network have very similar network structures. CHO et al [30] created the GRU network by combining the input and forgetting gates of the LSTM network and defining them as update gates, while adding reset gates. Compared to LSTM, GRU has fewer hyperparameters and is less computationally intensive. Its structure is shown in Fig 3. More detailed introduction of GRU algorithm can be found in CHO et al.

The time step, neuron, epoch, and optimization function for the GRU model training and prediction process are the same as for the LSTM model training and prediction process.

#### 2.2.3 One step ahead rolling forecast

In reality, new daily outpatient observations are available every day and are used to predict the next day’s outpatient volume. The rolling forecast scenario, also known as walk-forward model validation, is the same. The method combines time steps to obtain observations from the test set and then provides the values to the next time step for prediction [31]. It also means that the prediction is made by the time step. With rolling forecasts, we can keep pace with changes in outpatient volumes and quickly adjust outpatient resources. Therefore, the method will make the predictions more accurate.

#### 2.2.4 Evaluation indicators

The performance of the forecasting model is calculated using the Mean Absolute Error (MAE), Root Mean Squared Error (RMSE) and the coefficient of determination(*R*^2^).

$$MAE=\frac{1}{n}\sum_{i=1}^{n}\left|{\hat{y}}_{i}-y_{i}\right|,RMSE=\sqrt{\frac{\sum_{i=1}^{n}{\left({\hat{y}}_{i}-y_{i}\right)}^{2}}{n}},R^{2}=1-\frac{\sum_{i=1}^{n}{\left({y_{i}-\hat{y}}_{i}\right)}^{2}}{\sum_{i=1}^{n}{\left(y_{i}-\overset{-}{y}\right)}^{2}}.$$

Where *y*_*i*_ is the observed daily incidence of conjunctivitis on the *i*(*i* = 1, …, *n*) day, ${\hat{y}}_{i}$ is the predict daily number of outpatient visits, and the $\bar{y}$ is the mean value of the observed number of outpatient visits. If the values of RMSE and MAE are smaller, then the prediction error is smaller and the model is more accurate. The *R*^2^ value range is [0, 1].

## 3. Results

### 3.1 Results for LSTM and GRU models

The daily outpatient visits for conjunctivitis at the First Affiliated Hospital of Xinjiang Medical University from January 2017 to November 2019 were used as the training data set, and the data from December 2019 were used as the test set to build the prediction model. Ten alternative LSTM models and ten GRU models are listed in Tables 1 and 2. The results show that in the training set, Adam’s lstm model with 128 neurons has an RMSE of 0.99 for the training set, while RMSProp’s model with 64 neurons and GRU has an RMSE of 0.98 for the training set, and the optimal R of the LSTM and GRU models are consistent. The model with 32 neurons and the Adam of LSTM had the lowest RMSE for test set (RMSE = 2.86) in comparison with the models using other parameters. The model with 8 neurons and the RMSprop of GRU had the lowest RMSE for the test set (RMSE = 2.60) in comparison with models using other parameters.

**Table 1:** Comparison of the LSTM and GRU models in the training set.

Model		Time Steps	Neurons	Epochs	optimizer	RMSE
LSTM	1	7	128	250	Adam	0.99
	2	7	64	250	Adam	1.04
	3	7	72	250	Adam	1.17
	4	7	128	200	Adam	1.19
	5	7	64	200	Adam	1.54
	6	7	72	200	Adam	1.56
	7	7	72	150	Adam	1.82
	8	7	32	250	Adam	1.82
	9	7	128	150	Adam	1.87
	10	7	32	200	Adam	2.16
GRU	1	180	64	250	RMSProp	0.98
	2	180	72	200	RMSProp	1.03
	3	180	64	200	RMSProp	1.13
	4	180	32	250	RMSProp	1.15
	5	60	64	250	RMSProp	1.21
	6	30	64	250	RMSProp	1.24
	7	60	72	250	RMSProp	1.24
	8	60	72	200	RMSProp	1.32
	9	60	128	250	RMSProp	1.32
	10	30	64	200	RMSProp	1.38

**Table 2:** Comparison of the LSTM and GRU models in the validation set.

Model		Time Steps	Neurons	Epochs	optimizer	RMSE
LSTM	1	30	64	250	Adam	2.86
	2	30	128	150	Adam	2.93
	3	180	8	150	Adam	3.00
	4	30	32	150	Adam	3.03
	5	60	128	150	Adam	3.06
	6	30	8	200	Adam	3.07
	7	7	64	100	Adam	3.12
	8	60	8	250	Adam	3.13
	9	7	72	250	RMSprop	3.15
	10	60	128	250	Adam	3.16
GRU	1	7	8	200	RMSprop	2.60
	2	30	8	150	Adam	2.90
	3	7	16	250	Adam	3.00
	4	7	16	100	RMSprop	3.03
	5	30	8	200	Adam	3.12
	6	60	128	50	RMSprop	3.26
	7	7	64	100	Adam	3.28
	8	30	16	100	Adam	3.28
	9	30	64	250	Adam	3.29
	10	60	128	200	Adam	3.32

### 3.2 Prediction performance comparison

**Fig 4:**
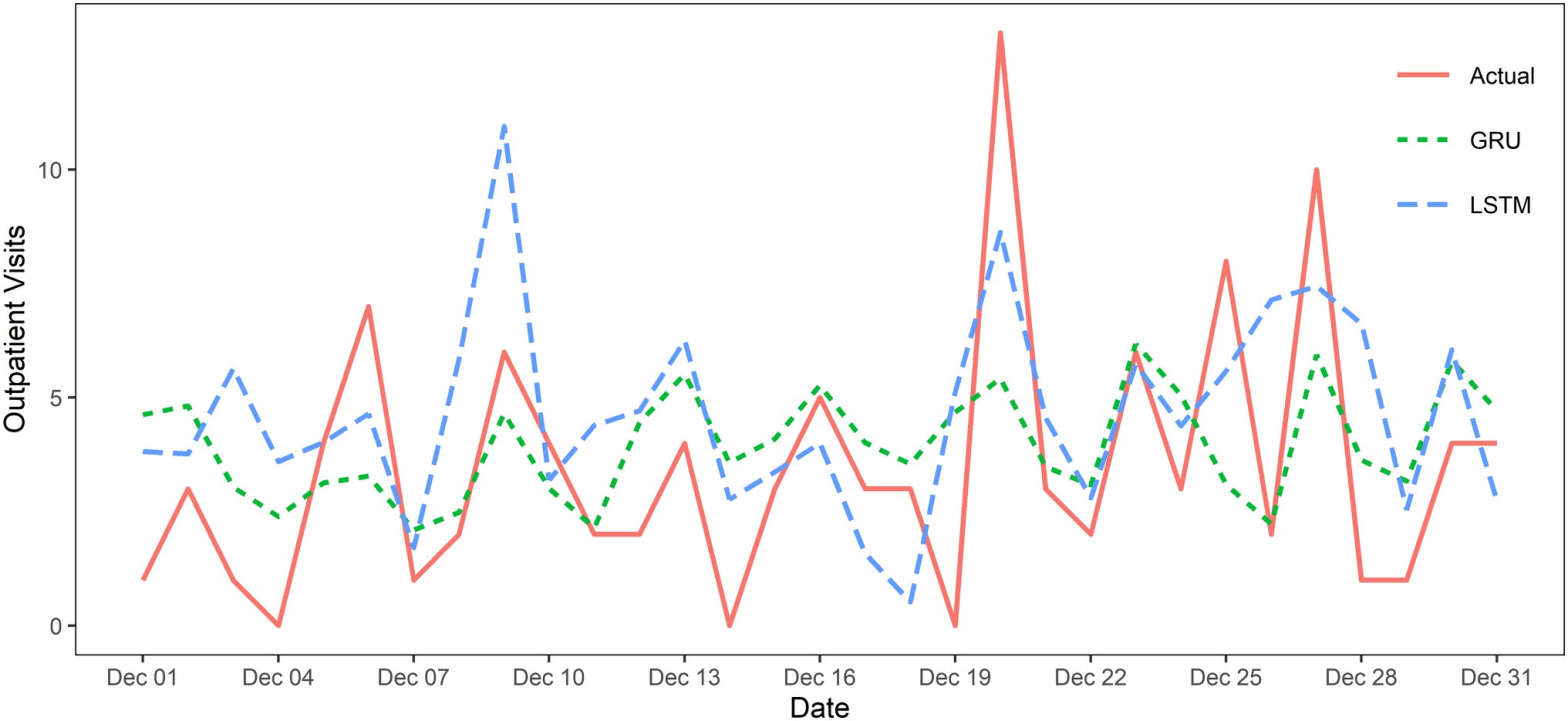
The actual daily incidence of conjunctivitis and values predicted by the two models in December 2019.

The prediction outputs are shown in Table 3 and Fig 4. Of the two models, the GRU model was better at predicting the number of outpatient visits for conjunctivitis over the next 31 days, with the smallest values for RMSE (2.60), MAE (1.99) and *R*^2^ (0.81). Compared to the LSTM model, the MAE accuracy of the GRU model is improved by 9.09%, the RMSE accuracy is improved by 16.74%, and the *R*^2^ accuracy is improved by 15.71%. Both metrics improved by a certain percentage point. That is, the GRU model is more accurate and precise in its predictions.

**Table 3:** The forecasting performance of the two models.

Model	RMSE	MAE	*R* ^2^
LSTM	2.86	2.39	0.70
GRU	2.60	1.99	0.81

## 4. Discussion

In this paper, we compare the effects of the LSTM and GRU prediction models on the number of conjunctivitis outpatient visits at the First Affiliated Hospital of Xinjiang Medical University. The results show that GRU is a better predictor and can provide a reference model for hospitals to predict outpatient visits. The finding that GRU achieved higher accuracy than LSTM is also consistent with previous studies [13,32–34].

There are a number of important parameters that need to be considered in the LSTM and GRU models when making predictions. The values of these parameters affect the model’s fit, training time, generalization ability, etc. In order to make the results comparable, the parameters in both models have been adjusted equally in this paper. Four different combinations of parameters were evaluated. These parameters were the time steps, the number of neurons, the number of training epochs, and the optimizer. The results show that the GRU model is the best model when the time step is 7, the neuron is 8, the epoch is 200 and the optimizer is RMSprop. Performance gets worse when the number of cells is less than 8, suggesting that too few units may lead to severe underfitting. But more cells do not necessarily lead to better results either, again due to phenomena such as overfitting. Within the epoch range set in this paper, the performance of the model improves as the training epoch gets larger. Such a change is consistent with the findings of Zhang et al [15]. The three compared optimizers were Adam SGD and RMSprop, Both RMSprop and Adam are optimization algorithms for SGD, RMSprop can solve the problem of sharply declining learning rates. Adam adds bias-correction and momentum to RMSprop. RMSprop and Adam are very similar algorithms and perform well in similar situations [35,36]. It can be seen from Table 1 that the results of the Adam and RMSprop optimizers in this paper do not differ significantly and they outperform SGD. The parameters were selected based on the value of the RMSE and the GRU model with the lowest RMSE on the test set was selected as the most optimal model in this study.

In conclusion, this paper suggests the use of the GRU method for predicting conjunctivitis data in the outpatient ophthalmology clinic of the First Affiliated Hospital of Xinjiang Medical University, and the method may provide a reference model that can be used to predict outpatient visits to the hospital. It also provides support for the management and allocation of medical resources, the arrangement of medical and nursing staff, and the study of consultation pathways; it provides an important theoretical basis for intelligent, detailed, and efficient hospital treatment.

Admittedly, this study has some limitations. The incidence of conjunctivitis is affected by air pollution, natural environment, and socioeconomic factors, however, due to data availability, and the focus on time series, these factors were not considered in this study. The next step in the study will be to incorporate more factors influencing outpatient volume, such as holidays, air quality, weather, etc., into the prediction model. Better integrated prediction models will be built to provide more accurate predictions in order to further delve into the practical applications of medical time-series data.

## Supporting information

S1 Data (CSV)
